# Review of Structural Health Monitoring Methods Regarding a Multi-Sensor Approach for Damage Assessment of Metal and Composite Structures

**DOI:** 10.3390/s20030826

**Published:** 2020-02-04

**Authors:** Christoph Kralovec, Martin Schagerl

**Affiliations:** 1 Institute of Structural Lightweight Design, Johannes Kepler University Linz, 4040 Linz, Austria; martin.schagerl@jku.at; 2 Christian Doppler Laboratory for Structural Strength Control of Lightweight Constructions, Johannes Kepler University Linz, 4040 Linz, Austria

**Keywords:** multi-sensor, data fusion, composite, metal, electro-mechanical impedance, guided wave, electrical impedance tomography, carbon nanotube, strain-based SHM

## Abstract

Structural health monitoring (SHM) is the continuous on-board monitoring of a structure’s condition during operation by integrated systems of sensors. SHM is believed to have the potential to increase the safety of the structure while reducing its deadweight and downtime. Numerous SHM methods exist that allow the observation and assessment of different damages of different kinds of structures. Recently data fusion on different levels has been getting attention for joint damage evaluation by different SHM methods to achieve increased assessment accuracy and reliability. However, little attention is given to the question of which SHM methods are promising to combine. The current article addresses this issue by demonstrating the theoretical capabilities of a number of prominent SHM methods by comparing their fundamental physical models to the actual effects of damage on metal and composite structures. Furthermore, an overview of the state-of-the-art damage assessment concepts for different levels of SHM is given. As a result, dynamic SHM methods using ultrasonic waves and vibrations appear to be very powerful but suffer from their sensitivity to environmental influences. Combining such dynamic methods with static strain-based or conductivity-based methods and with additional sensors for environmental entities might yield a robust multi-sensor SHM approach. For demonstration, a potent system of sensors is defined and a possible joint data evaluation scheme for a multi-sensor SHM approach is presented.

## 1. Introduction

Economic and ecological requirements of modern industries call for highly optimized lightweight structures. This is particularly true for the transportation industries where a vehicle’s deadweight and downtime should be minimized at the highest structural reliability. Uncertainties, e.g., true loading and material properties, environmental influences, or misuse, yield over-designed structures and the need for safety-related inspections (downtime). To address these uncertainties, structural health monitoring (SHM) was introduced in the 1990s as a derivative of non-destructive testing (NDT) [1]. SHM is the continuous, on-board monitoring of a structure during operation by integrated systems of sensors. Based on the information provided, SHM can be classified into five levels [2]: Level 1, detection of a damage; Level 2, localization of a damage; Level 3, quantification of a damage; Level 4, typification of a damage; and Level 5, assessment of the structures integrity. State-of-the-art SHM methods readily achieve up to Level 4. To advance today’s scheduled inspection-based maintenance into a condition-based or predictive maintenance, the assessment of the structure’s integrity is inevitable. However, thus far, only concepts exist for Level 5 damage assessment [3,4]. At the authors’ research laboratory, a comprehensive predictive maintenance concept for lightweight structures is under development that combines the following disciplines for instantaneous and predictive damage evaluation:

- Application of SHM, loads and usage monitoring for flaw indication and monitoring
- Structural analysis to assess the effects of flaws on structural functions and strength
- Implementation of condition- and prediction- based maintenance under consideration of regulation and safety standards

The current article presents a survey of the capabilities of SHM methods and their systematic and advantageous combination under this interdisciplinary aspect.

However, different physical effects are used to assess potential damage by numerous evaluation methods. SHM methods can, on the one hand, be classified into active methods, i.e., the observed structure is excited and its response to it measured (e.g., guided waves method [5,6,7,8]), and passive methods, i.e., operational loads or damage initiation directly cause the measured signal (e.g., acoustic emission [9]). On the other hand, SHM methods can also be classified as static methods, i.e., when static states are evaluated (e.g., neutral axis method [10] or electrical impedance tomography methods [11,12,13,14]), and as dynamic methods, i.e., when dynamic events are evaluated over time (e.g., electromechanical impedance method [2,15,16,17,18]). However, damage cannot be measured directly. Therefore, SHM systems monitor and evaluate the physical properties of a structure based on so-called damage features and damage indicators. A damage feature is a characteristic in the measurement data that can be assigned and is sensitive to the effect of damage on local or global properties of the observed structure. A damage indicator is a quantity that is used to evaluate a damage feature. Such a damage indicator is directly measured or calculated from the measured entities, and, thus, directly correlates with the presence and extent of the damage (e.g., guided wave time-of-flight (ToF) [19] or local static strain [20]). The assignment of damage features and indicators to a change in the structure is data- or model-based (e.g., guided wave triangulation for localization [19] and scattering pattern for identification [6]). However, single SHM methods and their damage assessment features are hardly able to reliably monitor all types of damage at every location of a given structure [21]. Furthermore, different SHM methods have different potentials to evaluate certain damages as it is advantageous to measure structural changes by most directly related features. Different types of damages effect different structural properties, and, thus, the combined use of various SHM methods and their features is better for an accurate and reliable evaluation [21]. Furthermore, uncertain environmental influences typically corrupt the evaluation and need to be compensated or identified. As a consequence, the fusion of multiple physical entities by multi-sensor approaches receives increasing attention in the scientific community. In 2018, e.g., the International Journal of Distributed Sensor Networks published a special collection dedicated to the topic of multi-sensor data for SHM [22]. Multi-sensor data fusion can be understood as either a fusion of homogenous sensor data (same sensor, respective data type, and physical entities; e.g., guided wave triangulation by three or more transducers [19]) or as a fusion of heterogeneous sensor data (different sensor, respective data types, and physical entities; e.g., neutral axis identification with temperature compensation by a Kalman filter [10]). However, the present contribution deals with the potential advantages of heterogeneous multi-sensor data fusion for SHM, with particular emphasis on the fusion of different SHM methods.

## 2. Data Fusion Overview

Data fusion techniques combine data from multiple sensors with information from an associated database to achieve more conclusions on a potential damage than a single sensor would yield (e.g., the fusion of three guided wave ToF features to achieve damage localization [19]). At the same time, data fusion techniques improve the accuracy and reliability of these conclusions [23,24]. Data fusion can be done at three different levels [23]:

1. Raw data fusion level: Multi-sensor data can be directly evaluated or combined to a sensitive feature for, e.g., detection, localization, quantification, and typification of damage, if the same physical phenomenon is measured by, e.g., classic detection and estimation methods (e.g., Kalman filter).
2. Feature data fusion level: Several representative damage indicators (of different damage features) are extracted from multi-sensor data and collected in a vector, which is evaluated by, e.g., pattern recognition approaches based on neural networks, clustering algorithms, or template methods.
3. Decision level fusion: Evaluation results from different damage assessment methods, e.g., detection, localization, quantification, and typification, are used for joint evaluation and decisions on consequences by, e.g., weighted decision methods (voting techniques), classical inference, Bayesian inference, and evidence theory (Dempster–Shafer method).

Building a data fusion system for particular applications, e.g., the assessment of structural damage, includes the following questions [23]:

- Which SHM assessment methods are appropriate and optimal for the considered structure and its damages of interest?
- What are the sensitive features for damage assessment?
- How should the data be fused (fusion system architecture)?
- What is the accuracy and reliability of the data fusion results?
- What are the environmental influences on the sensor data and their fusion-based evaluation?
- What is the range of operation of the defined data fusion architecture, i.e., within which boundary values (of structural data) does fusion improve the result?
- How can the data fusion accuracy and reliability be optimized dynamically?

However, damages are measured through their effects on specific structural properties. Thus, the optimal selection of one or more appropriate SHM assessment methods necessitates the knowledge of the structural properties that are most significantly influenced by the damages of interest. To quantify the interaction of the mechanical structure and the considered damages requires a profound analysis. Table 1 gives an overview (based on literature and experience of the authors’ research group) of different damage types in metal and composite structures and their qualitative influence on the local mechanical properties. Metals considered here are aluminum alloys, titanium alloys, and steels. Considered composites are particularly laminated carbon or glass fiber reinforced polymers (CFRP or GFRP, respectively).

Furthermore, features in the sensor data have to be found and used that allow concluding on, e.g., existence, location, extent, or type of a damage (cf. Section 3). To realize efficient SHM and optimally use the available information about the health state of a structure, the data fusion architecture governs the data flow within a damage assessment process. This architecture can also contain different levels of SHM, consecutively assessing the existence, location, extent, and type of a possible damage. Various damage assessment methods, their features, and their derived indicators might be more appropriate at different SHM levels (e.g., guided wave damage metric for existence, ToF for localization, and wave scattering patterns for identification; cf. Section 3). For the data fusion architecture, it is crucial to know accuracy and reliability of the different fusion levels, raw data fusion, feature fusion, and decision fusion. This is strongly related to the back-calculation from damage assessment features to structural changes due to damage. For SHM applications, this inverse problem is typically ill-conditioned, and, thus, very prone to uncertainties in the sensor data, such as measurement noise or environmental influences. This prevents a unique and robust interpretation. Table 2 shows numerous examples of environmental influences on different local mechanical properties and their qualitative effect (based on literature and experience of the authors’ research group). This multitude of influencing factors shows the difficulty of SHM to find features that allow a unique and robust damage assessment.

Data fusion can increase the reliability of the evaluation further by, e.g., obtaining an improved estimate of a physical phenomena via redundant observations or allowing a more accurate model to assess a damage due to multiple data entities (e.g., strain and temperature) [10,23]. However, the fusion of a very scattered and unreliable dataset with an appropriate dataset can also spoil the combined assessment result. In this case, it is better to rely only on the better dataset. Thus, operation ranges for the defined data fusion architecture and its dynamic optimization are needed to guarantee the improvement of the damage assessment due to data fusion.

## 3. Fundamental SHM Methods

The basic information for the development of an appropriate multi-sensor SHM approach for a given structure and its potential damages are the capabilities of the various available standalone SHM methods. These capabilities are basically given by the governing equations of the methods and the sensitivity of their physical parameters to damage. However, it is not only required to find the most sensitive multi-sensor approach. It is also important to find an approach that is very robust against uncertain environmental influences. In the following, a brief overview on the fundamentals of some of the most common SHM methods and their capabilities and drawbacks is given.

### 3.1. Static Strain Measurements with Fiber Optical Sensors (FOS)

The assessment of a structure’s mechanical behavior by strain measurements has a long history [4]. Due to their local nature, strain-based techniques have been traditionally used for fatigue monitoring at known hotspots on the structure. The standard sensor for such applications is the strain gauge. However, during the last two decades, the possible applications of strain measurements have exploded because of the advent of fiber optical sensors (FOS) and their distinctive advantages, such as high sensitivity, immunity to electro-magnetic interference, durability, ability to multiplex and distributed sensing, and potential to be embedded in the structure [33]. Therefore, currently, the scientific and industrial communities attribute the FOS technology the highest potential for continuous real-time monitoring of, e.g., aircraft structures [33].

Strain-based SHM methods can be classified into frequency and time domain techniques. Static strain measurements are done in the time domain. The time domain methods are, e.g., rain-flow counting algorithms for fatigue monitoring or other techniques for measuring local strain in known hotspots [34]. For more global monitoring, the new trend is to use neural networks and machine learning algorithms to evaluate correlations between strain sensors at different locations [35]. Significant difference between the estimated strain calculated from a model and the measured strain is attributed to a potential damage. Thus, strain measurements allow the assessment of every damage that changes the local load path (strain state) in a structure for a given loading (e.g., crack in composite or metal components) or that yields residual strains after a damaging event (e.g., impact in composite or plastic deformation of metal components). However, damages such as delaminations grown from manufacturing defects that only influence the strain state when high loads are applied (which forces the delamination to buckle) are difficult to detect. Furthermore, by a sufficiently dense sensor array, detection, localization, and size estimation of damage can be done but the identification of the damage type (SHM Level 4) has not been shown yet.

**Figure 1:**
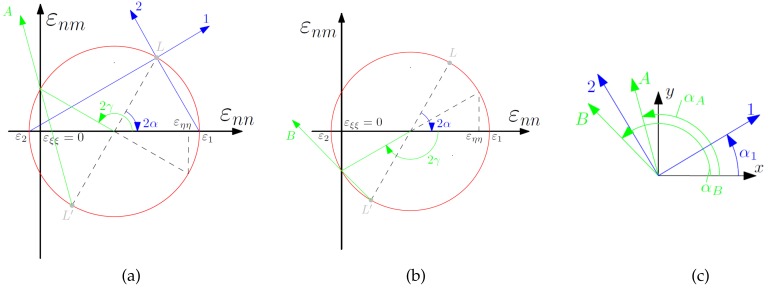
(**a**,**b**) Mohr’s circle for plane strain with zero-strain directions A and B; and (**c**) principal strain directions and zero-strain directions illustrated at the considered strain location [38].

However, to realize efficient strain-based SHM by a small sensor array, sensitive damage assessment indicators, e.g., the neutral axis location [36] or the zero-strain trajectory (ZST) [37], are needed. The latter method was developed at the authors’ research group and uses strain measurements along nominal zero-strain trajectories as damage sensitive indicators for the assessment of damages. Figure 1 illustrates the basic principle of the ZST method by Mohr’s strain circle for a considered arbitrary plane strain state. If the principal strains of a two-dimensional plane strain state have opposite sign, i.e., if the principal strains are tensile and compressive, directions exist where no normal strain can be measured. Connecting such points on a structures surface, so-called zero-strain trajectories can be drawn. Accordingly, the normal strains $\varepsilon_{\xi \xi }$ along these trajectories vanish, i.e., a FOS along a ZST of a structure does not show any strain for the load case (strain state) considered. If a change in the strain state of the considered load case occurs, e.g., due to a change in the load path as a result of damage, then the measured strain along the whole trajectory will change from zero to a finite value, showing thereby a most significant change in signal. This information allows the evaluation of the global strain state (load path changes effect the whole structure) and local strain state (close to a damage the effect is stronger) for damage assessment. The trajectories themselves can be found with model- or data-based methods and locally represent an optimal sensor placement. Moreover, the ZST method enables the use of the 1D-strain measurement of a fiber optical sensor (FOS) along a defined ZST for the assessment of a 2D-strain state.

**Figure 2:**
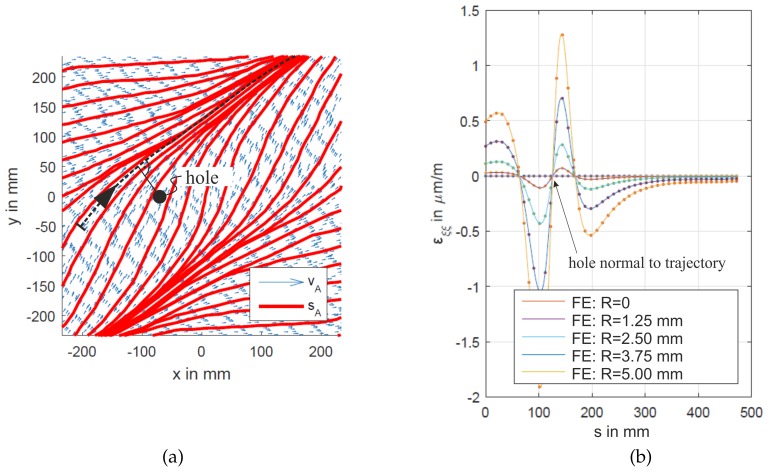
(**a**) Plate with zero-strain trajectories (direction A, red) and considered hole position (black circle); and (**b**) evaluated ZST (black dotted) for strain along black dotted ZST for increasing hole radius (R=0…5mm) [38].

The initial derivation of this novel method was done within a bachelor’s [39] and two Master’s theses [38,40], where it was demonstrated numerically and experimentally by a buckling plate with dimensions (in mm) 500×500×2. Figure 2 presents the numerical results of Riedl [38]. Figure 2a shows a set of ZST (direction A, compare Figure 1a) for the vertically loaded and buckled plate (simply supported at x=±250mm and clamped at y=±250mm). Figure 2b shows the strain $\varepsilon_{\xi \xi }$ along the curvilinear coordinate *s* of black dotted ZST (cf. Figure 2a) for a varying damage (hole) size, indicating the location (between min($\varepsilon_{\xi \xi }$) and max($\varepsilon_{\xi \xi }$)) and revealing a monotonous relation between the hole size and the measured extreme values max$\left|\varepsilon_{\xi \xi }\right|$. Thus, the evaluation of local strains along a ZST shows high potential for SHM Level 2 (localization) and Level 3 (conclusion on size) damage assessment. Furthermore, Figure 2b clearly shows the sensitivity of the ZST method to a change in the load path (due to damage), as for the considered ZST strains can also be calculated far from the actual damage.

Experiments confirm in principal all these results. However, experimental results highly suffer from plate and boundary support imperfections and are expected to be prone to environmental uncertainties, e.g., temperature, moisture, or misalignment in load introduction. Furthermore, digital image correlation (DIC) used for complementary spatial strain measurement in experiments shows a high signal-to-noise ratio for the—very small—strains and strain changes, which challenges execution and interpretation of ZST measurements.

### 3.2. Conductivity Measurements with Electrical Impedance Tomography (EIT)

Electrical impedance tomography (EIT) was developed as a method for noninvasive medical imaging of parts of the human body. It uses measurements of the electrical potential *V* at the boundary of a conductive body Ω of interest, which is supplied by different known electrical fields to conclude on its in-plane spatial conductivity, yielding the two-dimensional tomographic image. If there is no additional current source within the considered conductive body Ω, the sum of all current densities yields

(1) ∇·σ∇u=0,

where σ is the bodies conductivity, *u* is its scalar voltage potential, and, thus, ∇u yields the applied electrical field. If the conductivity is considered isotropic, i.e. σ is a scalar, then the the electrical potential within the body can be directly solved, yielding the boundary voltage potential $V=\nabla u\cdot \vec{n}$ of Ω by the boundary normal vector $\vec{n}$. Conversely, this boundary voltage potential *V* can be measured by an array of electrodes, allowing to do the inverse back-calculation of the bodies scalar conductivity σ by Equation (1) and considering the electrode contact resistances. The inverse back-calculation is done by finite element EIT solvers, which typically iteratively find the conductivity σ via least-squares estimation of measured boundary voltage responses to pre-defined current injection patterns. However, a much faster estimation approach, applied at the authors’ research group, is the one-step inverse method proposed by Adler [41]. It estimates the linearized solution by the maximum a posteriori (MAP) method and yields the conductivity change by

(2) $$\frac{\Delta \sigma }{\sigma_{0}}={\left(H^{T}WH+\lambda R\right)}^{-1}H^{T}W\frac{\Delta V}{V_{0}}\;,$$

where $\sigma_{0}$ is the conductivity at the pristine state of the body Ω and $V_{0}$ the boundary voltage gained from the baseline measurement. Δσ and ΔV are the conductivity and the boundary voltage changes, respectively, between pristine and damaged state of the body Ω. Furthermore, *H* denotes the sensitivity matrix that linearly transforms the change in conductivity to the boundary voltage response. The transformation is stabilized by the weighting matrix *W* (considers measurement noise), the regulation matrix *R* (compensates the ill-posed nature of the inverse problem by smoothing), and the hyperparameter λ (controls the smoothing). Different regularization methods can be used to define H,W, and *R* [42], but, for the selection of λ, a trial and error approach is still standard, although it strongly influences the reconstruction result [43,44]. However, the tomographic reconstruction of conductivity changes can theoretically be applied to any conductive body. For damage assessment, the changes in the conductivity of the observed body are used. This requires a baseline measurement, and, furthermore, limits the EIT to damages that cause a conductivity change. Additionally, the measurement instrumentation (including current source, voltmeter, contacting electrodes, cables, etc.) and their accuracy strongly influences the results as the conductivity changes are typically very small. To overcome this issue, the conductivity of a monitored body needs to be in a proper order of magnitude. Very small conductivity, e.g., glass fiber reinforced plastics, would need high voltages for current injection and is therefore not suitable. Very high conductivity, e.g., aluminum, result in extremely small changes of the electrical potential at the boundary electrodes due to strain or even small cracks. Therefore, today, EIT as SHM method for structural components is particularly interesting for two different applications, which are outlined in the following sections.

#### 3.2.1. EIT with Conductive Surface Layers

Conductive surface layers are thin polymer films doped with a conductive filler, e.g., carbon nanotubes (CNT) [44,45,46] or graphene [47]. Their conductivity σ or resistivity $\rho =\sigma^{-1}$, respectively, can be tailored to the application. Moreover, they show measurable conductivity changes due to, e.g., strain, pressure or presence of oxygen [44,45,46,48]. The use of a conductive surface layer also allows monitoring bodies with very low or high conductivity (integrating additionally an isolating layer). Due to their superior conductivity properties and elastoresistivity (i.e., change of the material’s resistivity due to applied strain), these layers not only allow the reconstruction of failure locations (damage that results in cracks in the surface layer) but also the reconstruction of strain states. Thus, they they are also applicable for internal damages that yield residual strain (e.g., impact damage) or stress/strain concentrations on the surface (under loading condition, e.g., larger internal fiber or matrix cracks) or for internal damages in adhesive joints [12,13,27,49].

**Figure 3:**
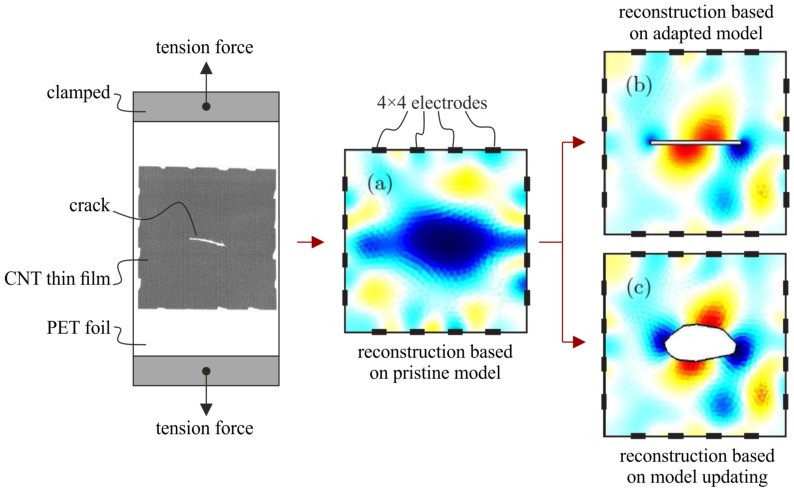
CNT embedded thin film attached to a PET tension test specimen with crack (cut by knife) loaded in vertical direction (left) and conductivity reconstruction results (**a**–**c**) [27,49].

Figure 3 shows exemplarily the conductivity reconstruction results (Figure 3a–c) of a CNT embedded thin film attached to a PET tension test specimen with crack (Figure 3, left) loaded in vertical direction. Figure 3a represents the reconstruction of the conductivity change Δσ without knowing the existence of a damage. Such an image can be easily used for:

- detection (SHM Level 1), by definition of, e.g., a threshold for a statistically based metric M(Δσ);
- localization (SHM Level 2), by, e.g., considering the area of largest conductivity change max(Δσ); and
- size estimation (SHM Level 3), by, e.g., evaluation of the conductivity change rate ∇(Δσ) or definition of a critical threshold value $\Delta \sigma_{\mathrm{cr}}<\Delta \sigma$ for rupture by learning algorithms [49].

**Figure 4:**
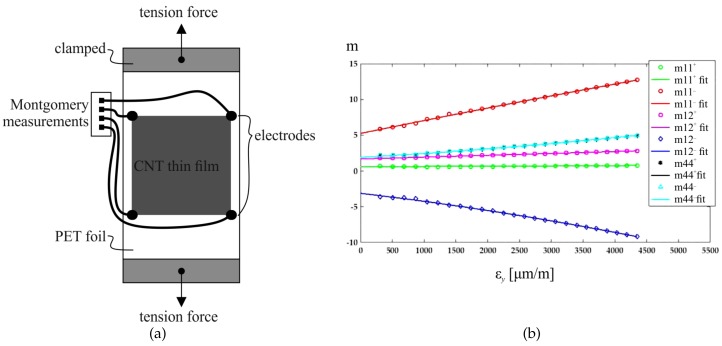
(**a**) PET tension test specimen with inkjet-printed CNT embedded thin film (black) and electrode layout for data acquisition for Montgomery method. (**b**) Strain direction dependent elastoresistivity tensor coefficients (+) and (−) and fitting function over increment of tensile strain applied to the test specimen [44].

Furthermore, by including the crack in the numerical model used for reconstruction, the strain concentrations at the crack tips can also be observed (cf. Figure 3b) [27]. However, this update of the numerical model would require the knowledge of the damage type and shape. Without this a priori knowledge, Figure 3c shows that a self-organizing map algorithm also yields acceptable results for damage shape and subsequent strain distribution [49]. The CNT embedded thin film’s elastoresistivity, which enables strain monitoring, is given by the tunneling effect of the CNT [44,50]. It is defined by:

(3) $$\begin{array}{c} \frac{\Delta \rho }{\rho }=m\cdot \varepsilon \;,\;\mathrm{respectively}\;\\ \frac{1}{\rho }\left(\begin{array}{c} \Delta \rho_{x}\\ \Delta \rho_{y}\\ \Delta \rho_{xy} \end{array}\right)=\left(\begin{array}{ccc} m_{11} & m_{12} & 0\\ m_{12} & m_{11} & 0\\ 0 & 0 & m_{44} \end{array}\right)\left(\begin{array}{c} \varepsilon_{x}\\ \varepsilon_{y}\\ \varepsilon_{xy} \end{array}\right) \end{array}$$

where Δρ is the spatial resistivity change tensor, ε is the in-plane strain tensor, and m is the considered material’s elastoresistivity tensor [44]. The strain direction dependent elastoresistivity components $m_{11}=m_{22},\;m_{12}$, and $m_{44}=\left(m_{11}-m_{12}\right)/2$ for isotropic materials can be found by, e.g., the Montgomery method and loading of the considered conductive thin film in two different directions [44]. Figure 4a presents a PET specimen with an applied inkjet-printed CNT embedded thin film and electrodes applied to the four corners for data acquisition for the Montgomery method. Rotating the inkjet-printed CNT embedded thin film on such a specimen for a second Montgomery measurement allows the back-calculation of the components of the elastoresistivity tensor m, as presented in Figure 4b.

However, there are indications that the unique reconstruction of the anisotropic resistivity coefficients $\Delta \rho_{x}$, $\Delta \rho_{y}$, and $\Delta \rho_{xy}$ with EIT is not possible [51]. Applying the classical approach given by Equation (2), the reconstruction only yields an (in some sense) equivalent scalar isotropic conductivity Δσ. Other issues arise with damages that influence in-plane loading paths only little and thus result in little strain and conductivity changes. A challenging example for this is a composite delamination that has grown from a manufacturing defect. Due to a sole delamination, in-plane loading paths only change slightly, and, moreover, only change if compression buckling of the delaminated plies occurs. Additionally, conductive thin films need high manufacturing quality and adequate isolation to the substrate [52]. Environmental influences on the surface layer’s conductivity (e.g., due to temperature, moisture, and electromagnetic radiation) and deviations caused by the measurement equipment itself (e.g., temperature and electrode resistance changes) are often in the same order as the effects of the damage. Thus, evaluation methods for a reliable real life application are an active area of current research.

#### 3.2.2. EIT with Conductive Structural Components

EIT can be applied directly to conductive structural components if their resistivity is in an adequate range, which is typically not true for the highly conductive metal components. CFRP components, as they are used in automotive and aeronautical industries, have a conductivity that allows the application of EIT directly [14,53,54]. Using the structural component itself has the big advantage that no additional sensor is needed, i.e., only the electrodes need to be applied. However, compared to conductive surface layers, the conductivity cannot be tailored easily. Furthermore, as the conductivity is predominantly given by the carbon fibers, the conductivity of layered CFRP is inherently anisotropic and inhomogeneous [14], making a unique reconstruction of the spatial conductivity by EIT according to mathematicians impossible [51]. Thus, reconstructions of an (in some sense) equivalent isotropic conductivity are from the beginning much more difficult to interpret than results with conductive and isotropic surface layers. Moreover, the EIT with conductive structural components suffers even more from environmental influences than the EIT with a conductive surface layer. Some prominent influences are caused by the measurement equipment and the quality of the electrode attachment that readily provoke conductivity changes in the order of the measurement. Therefore, electrodes that deliver unreliable data need to be identified and removed from the EIT reconstruction. A simple method to identify corrupted electrodes is to evaluate the standard deviation of multiple voltage change measurements SD(ΔV) for the same structural condition. Reducing the number of electrodes also reduces the spatial resolution of the reconstruction [55], and, thus, effects all levels of damage assessment including the reliability of the results. Particularly if the lost electrodes are locally accumulated, the reconstruction quality might be very low at this region. Nevertheless, reconstruction remains possible. A minimum number of electrodes used by researchers in experiments is eight [55,56]. Additional electrodes or a pattern with more measurements (for a given number of electrodes) theoretically improve the reconstruction result. The optimum number of electrodes is a trade-off between spatial resolution and the available equipment for measurement and computation, desired reconstruction and measurement time, and wiring effort [55,57].

**Figure 5:**
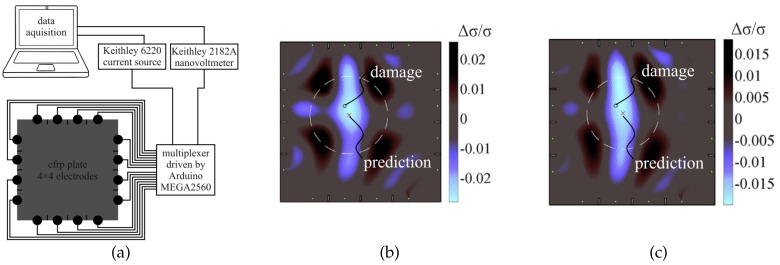
(**a**) Experimental setup: pristine plate with rivet-electrodes and measurement devices. (**b**) Reconstructed conductivity change from measurement for diagonal current injection pattern and a hyperparameter λ=0.0029. (**c**) Reconstructed conductivity change from 3D numerical simulation for diagonal current injection pattern and a hyperparameter λ=0.0029. The circle indicates the defect position (5-mm hole). The red cross is the center of gravity of the minimum one-forth amplitude set (white circle) [14].

However, experiments with a hole in a orthotropic multi-layer CFRP composite plate show the applicability of the EIT for damage detection and localization [14]. Figure 5 presents the experimental setup used (Figure 5a), the reconstruction of the spatial conductivity change Δσ from measurement results (Figure 5b), and the reconstruction of the spatial conductivity change Δσ from simulation results (Figure 5c).

First, the experimental results validate the detailed finite element model. Second, both measurement and simulation results clearly show the possibility to detect (SHM Level 1) and localize (SHM Level 2) a hole (5-mm diameter) in a CFRP plate with dimensions (in mm) 250×250×1.8. Damage size (SHM Level 3) and type (SHM Level 4) assessment might be possible in the future through large measurement- or model-based evaluation datasets or consideration of the CFRP material’s anisotropic conductivity. However, extensive test campaigns are not feasible and, to gather sufficient information on how measurement and simulation data are correlated, an even more detailed 3D model and advanced EIT algorithms would be required [14]. Unpublished results at the authors’ research group are promising, showing that barely visible impact damages can also be detected, localized, and roughly quantified by EIT in orthotropic multi-layer CFRP composite plates.

### 3.3. Vibration Analyses with Electro-Mechanical Impedance Method

The electro-mechanical impedance (EMI) method is a vibration-based structural inspection method. Its measure is the impedance of a E/M system, consisting of one [58,59] or more [60] piezoelectric wafer active sensors (PWAS) bonded to the mechanical structure of interest. The PWAS is used to excite the E/M system and at the same time to measure its frequency response in the form of the piezoelectric element’s impedance

(4) $$Z\left(\omega \right)=\frac{U(\omega )}{I(\omega )}\;,$$

where U(ω) is the amplitude of a harmonic voltage applied and I(ω) is the resulting and measured current at the PWAS for different angular frequencies ω=2πf. The harmonic voltage U(ω) excites the E/M system via the PWAS by the piezoelectric effect of expansion and contraction caused by the electrical field. The measured current I(ω) at the PWAS represents the response of the E/M system, which is directly related to the structural response. Consequently, a change in the impedance *Z* represents a change in the monitored mechanical structure, i.e. a damage. For the one-dimensional case, if damping and dynamics of the PWAS are neglected, the relation of the measured impedance and the dynamic response of the structure is given by [7]

(5) $$Z\left(\omega \right)=\frac{1}{j\omega C}{\left(1-\kappa_{31}^{2}\frac{k_{\mathrm{dyn}}\left(\omega \right)}{k_{p}+k_{\mathrm{dyn}}\left(\omega \right)}\right)}^{-1},$$

where $\kappa_{31}$ represents a non-dimensional material dependent coupling coefficient and j is the imaginary unit. The complex value of the impedance Z(ω) of an E/M system is defined by the electric impedance of the PWAS mainly given by its capacity *C* and the mechanical impedance of the system, basically characterized by the static stiffness of the PWAS $k_{p}$ and the dynamic stiffness of the monitored structure $k_{\mathrm{dyn}}\left(\omega \right)$, respectively. The latter can be expressed by

(6) $$k_{\mathrm{dyn}}\left(\omega \right)=-m\omega^{2}+jc\omega +k_{\mathrm{stat}}\;,$$

where *m* is the mass, *c* is the damping coefficient, and $k_{\mathrm{stat}}$ is the static stiffness of the considered structure. The static stiffness $k_{\mathrm{stat}}$ is a function of the elastic modulus *E*, Poisson’s ratio ν and the structure’s geometry, which we symbolize by a parameter *a*. Consequently, the EMI method and all other vibration-based SHM methods are sensitive to all kinds of structural changes related to material stiffness *E*, mass *m*, damping *c*, and geometry *a*, and, thus, theoretically applicable to the monitoring of all typical damage types of metal and composite structures (cf. Table 1).

However, due to the large number of structural properties considered and their sensitivity to environmental influences (cf. Table 2), a unique damage assessment is challenging. Furthermore, it is difficult to accurately predict structural vibration by, e.g., the Finite element (FE) method. This is particularly true for the high frequency vibration needed to assess small damages. To overcome these issues, the implementation of the EMI method as a SHM monitoring system is typically realized in two steps. First, the impedance spectra of the the pristine E/M system is identified over a wide frequency range by a so-called baseline measurement. Second, the impedance spectra of the E/M system is continuously monitored and compared to the baseline measurement throughout the whole operational life of the mechanical structure. Deviations of the impedance *Z* or admittance Y=1/Z characteristics are used as features for sensor self diagnosis and damage evaluation. Typically used features for self diagnosis of PWAS transducers are, e.g., the PWAS capacity change ΔC for monitoring of its possible rupture, the measured imaginary part of the admittance Im(*Y*), and new resonance frequencies that can be assigned to a freely vibrating PWAS for monitoring of the PWAS-structure bonding [7,61]. To evaluate the presence of a damage in the monitored structure, probabilistic neural networks can be applied to identify characteristics of the impedance spectra over a considered frequency range [17].

**Figure 6:**
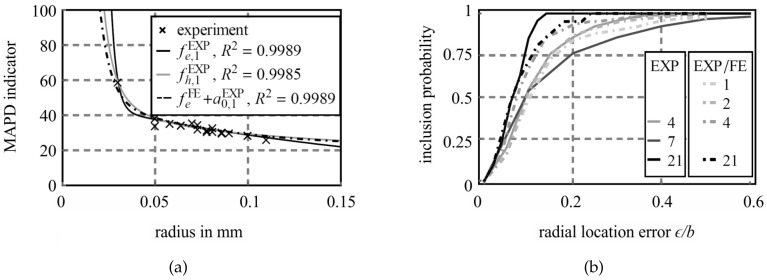
(**a**) MAPD damage indicator over distance *r* to the PWAS. The measurements are fitted with functions $f_{e,1}^{\mathrm{EXP}}=ae^{br}$, $f_{h,1}^{\mathrm{EXP}}=a\left|H_{b}^{\left(1)(r\right)}\right|$, and $f_{e,1}^{\mathrm{FE}}+a_{0,1}^{\mathrm{EXP}}$ from measurements and simulations. (**b**) Comparison of cumulative probability that true damage locations are enclosed within a radius ϵ to the data-based (EXP) and model-based (EXP and FE) predicted damage location [24].

The most basic method is to use statistically based metrics as damage indicators that simply reflect the difference between the initially measured impedance at the pristine structure and the current state of the structure for a defined frequency range. Typically, the real part of the impedance is evaluated by these metrics M(Re(Z)). Examples are the root-mean-square-deviation (RMSD) or the mean-absolute-percentage-deviation (MAPD) [7,15]. These damage metrics can furthermore be used to evaluate the distance of a detected damage to the PWAS transducer due to the effect of attenuation of the vibration with increasing distance, allowing thereby to localize a damage in a plate by triangulation [24,62]. Figure 6a shows the correlation of different attenuation functions with experimentally measured MAPD damage indicator for an aluminum plate of dimensions (in meter) 1.48×1.25×0.002 with magnets as movable artificial damages at a distance from the applied PWAS transducer. Furthermore, Figure 6a shows that fit functions $f_{e,1}^{\mathrm{FE}}$ found by numerical simulations yield a good approximation of experimental results if shifted by only one fit-parameter $a_{0,1}^{\mathrm{EXP}}$. Figure 6b shows that this allows a better localization reliability (inclusion probability) for a required accuracy level ϵ/b (radial location error ϵ normalized by PWAS distance *b*) at a reduced number of experimental measurements.

**Figure 7:**
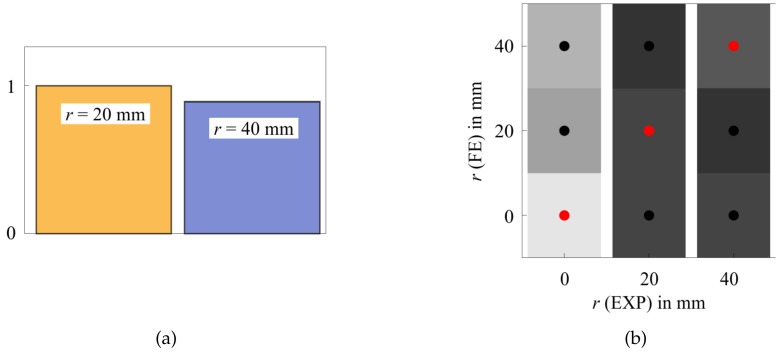
(**a**) Normalized MAPD damage metric indicators for 20-mm sandwich plates with different debonding sizes and frequency spectra Re(*Z*). (**b**) Comparison of MAPD damage metric indicators of sandwich plates with different debonding sizes *r* calculated for every combination of numerical (FE) and experimental (EXP) results for frequency spectra Re(*Z*) [15].

Furthermore, damage metrics can be used to conclude on the damage size by a pure data-based approach [7]. However, this can yield non-unique results for specific frequency ranges due to the overlap of resonance frequencies of different damage sizes [15]. This effect is demonstrated in Figure 7a. The considered specimens are circular aluminum sandwich panels with idealized debondings and with a PWAS transducer position at the center of the damage. Figure 7a shows the experimental MAPD damage indicator for a debonding size of radius r=20 mm and r=40 mm compared to the pristine state, refuting the assumption that the metric grows with increasing damage size. A model-based approach presented in [15] overcomes this problem by calculating damage metrics between parametric FE simulation results for a number of damage sizes (of the known damage type) and an experimental measurement with unknown size. The smallest signal deviation (represented by the damage metric) between model and experiment shall finally identify the size. Figure 7b presents the resulting comparison of MAPD damage indicators of Re(*Z*) for different combinations of experimentally measured and numerically simulated damage sizes. The shading represents the normalized damage indicator (light gray corresponds to a small value), allowing thereby to assign the best fitting FE model (red dots), and, thus, to identify the damage size.

Besides the presented analysis with damage metrics, researchers also applied probabilistic neural network algorithms [17,58,63] to detect, localize, and quantify damages. However, damage type identification (SHM Level 4) is a constant issue. Model-based approaches, such as the one presented above, are expected to reach this level, but have not been proved yet. Advancement might yield, e.g., the inclusion of nonlinear features such as higher harmonic response, due to contact acoustic nonlinearity. This would allow limiting the possible damage types to damages that have the potential to produce higher harmonic oscillation (all types of cracks) or not [16,18,64].

### 3.4. Ultrasonic Guided Waves (UGW)

The ultrasonic guided waves (UGW) method received a lot of attention in the SHM research community during the last decades [25,65]. This is because of the ability of ultrasonic waves to travel in thin-walled structures over long distances with little loss of energy [66] and its strong interaction with structural changes, i.e., potential damages, and cost-efficiency [66,67]. UGWs are elastic waves that are confined (guided) in structures by distinct boundaries. Such waves can be classified by their deformation modes. Guided waves that are in focus for SHM applications are:

- shear (horizontal and vertical);
- Lamb; and
- Rayleigh waves.

Shear waves—also called transverse waves as the movement of the medium is perpendicular to the direction of wave propagation—exist in two forms: the shear horizontal (SH) and the shear vertical (SV) waves. An application for SH waves is, e.g., under water inspection, as these waves are little attenuated by the surrounding liquid medium [67]. Lamb waves are guided between two parallel free surfaces, such as the lower and upper surface of an infinite plate. For real structural components, this is assumed to be given for a large ratio between in-plane dimension and thickness. Such waves propagate as superposition of symmetric and asymmetric modes. For small frequency-thickness products, only the fundamental symmetric S0 and asymmetric A0 wave propagate, allowing easier interpretation of measurement results [68]. For large frequency-thickness products, the superposition of symmetric and the asymmetric modes yields the transition to Rayleigh surface waves. Rayleigh waves propagate on free surfaces of structures, and, thus, are convenient for inspection of structural defects near to surfaces [28].

SH and in particular Lamb waves show little loss in amplitude when traveling over longer distances [69]. Therefore, since the early 1960s, many studies related to the application of Lamb waves for SHM in metallic and non-metallic structural components have been conducted [66].

For SHM applications, typically PWAS transducers are used to excite UGWs with frequencies within a defined frequency spectrum. Each wave is traveling with its own phase velocity. For example, the bending dominated A0 wave in an infinite plate travels with $c_{f}=\sqrt{E/2\rho_{s}\left(1+\nu \right)}$, where *E* is the elastic modulus, ν is the Poisson’s ratio, $\rho_{s}$ is the mass density, and ω=2πf is the angular frequency of the propagating wave. Together, these waves of different frequencies form a wave packet that travels with the group velocity $c_{g}$. However, as single waves travel at different wave speeds $c_{f}$, these packets are highly dispersive. In summary, the UGW method is advantageous for SHM application for two reasons:

- The propagation speed and attenuation (energy loss due to traveling) of UGWs depend on the structure’s material properties (elastic modulus, density, and damping) and geometrical properties (e.g., wall thickness, material phase transitions, micro (matrix) cracks, and surface roughness).
- The wave packets are reflected by sudden changes of these properties (e.g., due to cracks or corrosion in metal and cracks or delamination in composite).

The attenuation of traveling elastic waves can be described by an exponential decrease $a_{t}/a_{0}=e^{-\gamma (\omega )x}$, where $a_{t}$ is the wave amplitude after a distance *x*, $a_{0}$ is the initial wave amplitude, and γ(ω) is the frequency ω dependent attenuation coefficient that includes diffraction, reflection, diffusion, and absorption effects [62].

Furthermore, damages often provoke mode changes between the symmetric and asymmetric modes, e.g., a fundamental S0 wave is partly reflected as a fundamental A0 wave at a delamination. This multitude of interactions of UGWs with structural changes makes it a very versatile and sensitive SHM method. However, this property also yields the method’s drawbacks. For example, surface coatings can cause high attenuation. UGWs also interact sensitively with, e.g., dirt or material property changes due to uncertain environmental influences such as temperature or moisture [30,32]. The requirements on experimental equipment and setup are therefore high. The assessment of sensor measurement data is highly demanding, in particular as environmental influences or measurement noise corrupt the signals. Furthermore, analytical solutions only exist for a number of special cases (e.g., plate with hole) and simulations are very costly.

Commonly used UGW measurement techniques in SHM are the pitch–catch method (a PWAS transducer sends a burst signal that is collected by one or more other PWAS transducers) and the pulse–echo method (a PWAS transducer sends a burst signal that is collected by the same and possibly further PWAS transducers). Signal features used for damage assessment are changes in the signal’s intensity, energy, velocity shift, arrival time, amplitude, and impedance [66]. Self diagnosis of the sensors is typically done by EMI measurements (cf. Section 3.3) [7,70]. For the different levels of SHM, different signal features are considered.

#### 3.4.1. Damage Detection and Localization with UGW

For the pure detection of a damage (SHM Level 1), simple statistical damage metrics analyze the difference between the measured signal in the pristine state of the structure and in its actual state (when new wave packets are scattered at damages) [70].

**Figure 8:**
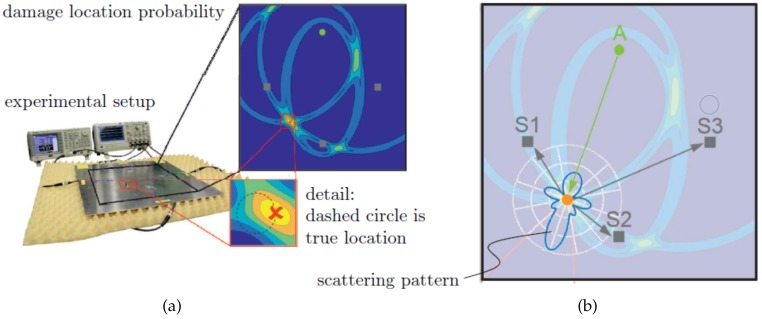
(**a**) UGW damage (magnet) localization in thin aluminum plate by ToF triangulation with one actuator (A) and three sensors (S). (**b**) Identification of damage by its characteristic frequency dependent scattering pattern evaluated at the three sensor position angles [71].

The localization of damages (SHM Level 2) is typically realized by triangulation, evaluating the ToF of new wave packets that are scattered at damages (e.g., the fusion of damage indicators by statistics, i.e., feature level fusion) [19]. An experiment of damage location by triangulation with three PWAS transducers is presented in Figure 8a. As specimen, a thin aluminum plate with magnets as movable artificial damages is used [71]. The ToF of wave packets is allocated by statistics, which yields the highest probability for damage location at the intersection of the three ellipses drawn around each actuator and sensor pair.

#### 3.4.2. Damage Identification with UGW

The identification of damage size and type (SHM Levels 3 and 4) needs more advanced methods. However, the simulation of traveling elastic waves and their interaction with potential damages in mechanical structures is numerically very demanding and time consuming. A promising model-based approach to address this issue was introduced by Giurgiutiu and Shen in 2016 [72]. Their so-called Combined Analytical Finite Element Approach (CAFA) uses an analytical model to efficiently predict the global wave propagation in undisturbed plate regions. Damages are only considered as point sources that reflect incoming waves according to a predefined transfer function. These transfer functions or scattering patterns are represented by wave damage interaction coefficients $C_{AA}$, where AA stands for the interaction between an incident A0 and the reflected A0 wave. Other coefficients can be found for every other wave mode interaction at a damage. The coefficients depend on the frequency *f* and sensing angle $\phi_{\mathrm{Sen}}$ (measured from incident wave propagation direction) and are calculated in the frequency domain by very efficient local FEM models. Once the location of a damage with respect to the position of the PWAS is known, a catalog of pre-calculated or pre-measured scattering patterns is consulted. Identification of damage size and type is possible, as every damage has its characteristic frequency dependent scattering pattern. Figure 8b presents exemplarily the numerically calculated and normalized scattering pattern (amplitude at 200 kHz) for a cylindrical steel obstacle bonded on a thin aluminum plate. The pattern is allocated at the position of the damage found by triangulation and oriented in direction of the actuating transducer.

**Figure 9:**
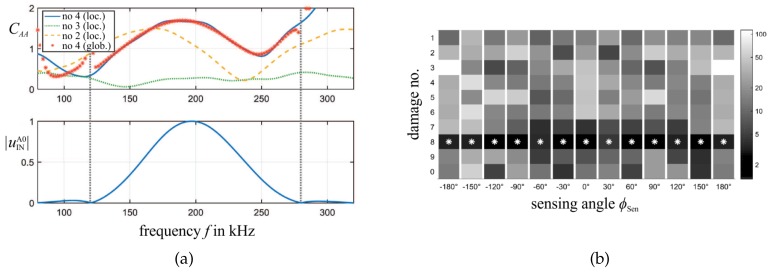
(**a**) (top) Comparison of the global results (asterisks) to the amplitude coefficients $C_{AA}$ taken from the WDIC catalog for different kind of damages in one sensing direction $\phi_{\mathrm{Sen}}=210^{\circ }$. (bottom) Normalized amplitude of the incident wave displacement $\left|u_{\mathrm{IN}}^{A0}\right|$. (**b**) Deviation metric M$\left(C_{AA}\right)$ of damage No. 8 (global transient model) to damages Nos. 1–10 (local steady-state models) (asterisks indicate smallest deviation, and thus the identified result) [71].

The applicability of this approach for SHM Level 4 identification is numerically demonstrated in Figure 9 by a thin aluminum plate with affixed steel obstacles of different shapes and different orientations. Thereby, it is shown that the sole consideration of the A0 wave amplitude has high identification capability readily for one PWAS sensor (i.e., at one scattering pattern direction) [71]. Figure 9a presents simulated (by a local steady-state and a global transient model) damage interaction coefficients $C_{AA}$ for the frequency range of a 200-kHz burst signal and the sensing angle $\phi_{\mathrm{Sen}}=210^{\circ }$.

**Figure 10:**
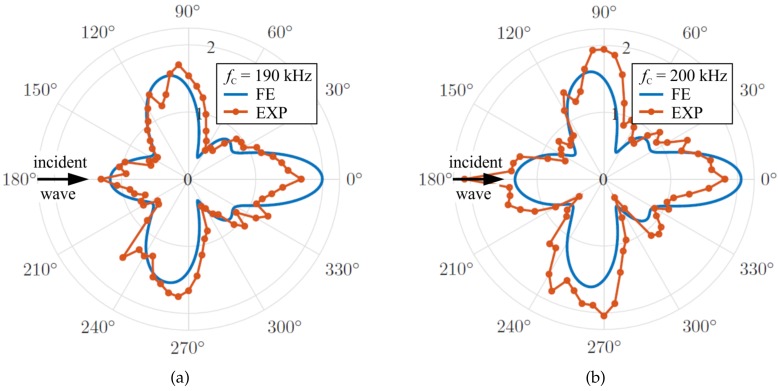
Comparison of numerically calculated (FE) and with laser scanning vibrometry measured (EXP) damage interaction coefficients $C_{AA}$ for burst center frequency: (**a**) $f_{c}=190$ kHz; and (**b**) $f_{c}\;=\;200$ kHz [74].

Both the global transient results (asterisks) and the local steady-state model results correlate very well for the same damage type but deviate over a wide frequency range for other damage types, orientations, and sizes. In this way, the approach can be used to identify damages. However, numerically calculated damage interaction coefficients $C_{AA}$ deviate from coefficients evaluated from laser scanning vibrometer measurements [73,74], as presented in Figure 10 for a cylindrical steel obstacle bonded to an aluminum plate (similar to the one depicted in Figure 8b).

Therefore, a reliable method for correlation of numerical and experimental results has to be found. In Figure 9b, the damage interaction coefficients of different damages are compared for different sensing angles $\phi_{\mathrm{Sen}}$ by a simple deviation metric M$\left(C_{AA}\right)$. The magnitude of this indicator allows identifying the damage, as illustrated by the level of gray-shading.

**Figure 11:**
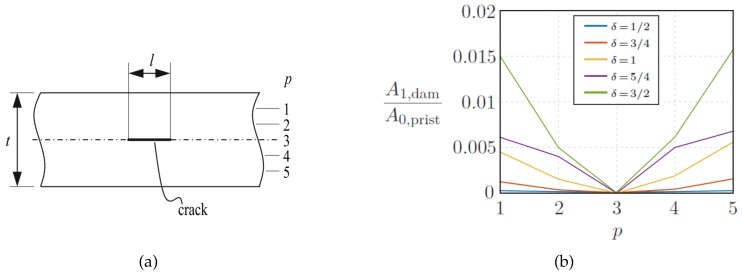
(**a**) Sub-surface crack with position *p* within thickness *t* and length *l* in an isotropic plate. (**b**) Higher harmonic amplitude ratio $A_{1,\mathrm{dam}}/A_{0,\mathrm{prist}}$ comparing wave packets in damaged and pristine conditions. The ratio is drawn for sub-surface cracks with different ratio δ=l/t and evaluated at a distance of 125 mm after the wave packet has passed the crack [26].

However, the method carries further identification potential by inclusion of, e.g., phase of the signal, symmetric modes, higher mode numbers, mode conversion, or possible nonlinear response. Particularly nonlinear features and corresponding mode conversions show high potential for the identification of delamination and sub-surface cracks [26,75,76,77], respectively. An example for sub-surface crack location and size correlation by evaluating nonlinear features of the signal is given in Figure 11. Figure 11a presents the investigated 2D model. Figure 11b shows the strong correlation of the crack location *p* and its size *l* with the amplitude of the first higher harmonic harmonic response $A_{1,\mathrm{dam}}$, which was excited by a burst of center frequency $f_{c}=260\;\mathrm{kHz}$.

However, the reliable implementation of such nonlinear approaches in real-world applications is still under research.

### 3.5. Summary of Damage Assessment Capabilities of SHM Methods

The capabilities of SHM methods are mainly given by the structural properties to which they are sensitive and if the extracted damage features can be back-calculated to information on existence, location, type, and size (SHM Levels 1–4) of a damage. Table 3 shows an incomplete summary of the influence of structural properties in SHM methods and the thereby reached SHM level. This current assessment is based on the explanations presented in Section 3.1, Section 3.2, Section 3.3 and Section 3.4.

The sensitivity to properties given in Table 3 can be directly compared to the property–damage assignment given in Table 1 and the environmental influences on local properties of mechanical structures given in Table 2. Considering the achievable and necessary level of damage assessment yields a rather straightforward selection of appropriate SHM methods for a multi-sensor SHM approach.

## 4. Multi-Sensor Approach to SHM of Metal and Composite Structures

A comprehensive and reliable SHM approach up to Level 4 damage assessment for both metal and composite structures and their numerous damage types can hardly be realized by a single state-of-the-art SHM method. This is in particular true because all SHM methods are prone to changing environmental influences and show different sensing capability and sensitivity to structural properties and structural changes (cf. Table 1, Table 2 and Table 3). Here, a multi-sensor approach can help to yield a more comprehensive, accurate, and reliable damage assessment result. To establish a multi-sensor SHM system, first, several SHM methods and their required network of sensors have to be selected that are capable of assessing the considered structural damages, and, second, a comprehensive data evaluation process needs to be defined to realize reliable damage assessment.

### 4.1. Selection of SHM Methods and Sensors

The selection of SHM methods directly interacts with the needed measurement entities and the required sensors. The applied sensor network design (sensor type and location, measurement equipment, etc.) is defined by, among others:

- optimal sensor positions for the intended measurement (excitation and measurement);
- robustness (measurement signal, equipment reliability, etc.);
- geometrical and physical constraints of the considered structure (volume, weight, curved surfaces, etc.);
- environmental constraints (areas prone to dirt, moisture, high temperature, etc.); and
- monetary constraints (sensor or measurement equipment).

However, the definitive design of the sensor network is an engineering task, which is not addressed in this article. Rather, a qualitative analysis for potential SHM methods and their sensors is given based on the theoretical capabilities (e.g., assessable damages or SHM level) of these methods. Recalling the state-of-the-art potential of the SHM methods presented in Section 3, full identification of a damage can only be achieved by an active dynamic method such as the UGW method.

Dynamic methods have the advantage of being very sensitive to many structural properties and their changes. For the same reason they are also prone to environmental influences on structural properties. Furthermore, the back-calculation of higher level damage features is complex, and, thus, becomes challenging for noisy measurement data with unknown environmental influences. As a consequence, measurement entities that are more directly related to specific structural changes should be included for a comprehensive and reliable multi-sensor SHM approach. This is, e.g., provided by static strain-based methods such as the ZST approach, where the structural mass and damping properties are not evaluated and many damage types directly yield changes in the measured surface strains (cf. Table 1). FOS have a comparatively high technological readiness level and allow applying the strain-based methods along a curve or even large areas by the use of a sensor grid. This also enables conclusions on location and size of damages. Furthermore, methods that might be less reliable can be used to complete the damage assessment or decrease the required sensor grid density. A method that does not need sensors at all by using the electrical properties of a conductive structure (e.g., CFRP) is the direct EIT. It allows a rather complete monitoring of location and size within the whole volume but is again more prone to environmental influences. Furthermore, the costly evaluation, required measurement equipment, and robust attachment of electrodes at the considered structure are still an issue of the direct EIT. However, in a combined usage with other SHM methods, the EIT-based damage assessment might already yield valuable results.

Another issue that can be addressed by a multi-sensor SHM approach is the ability to reduce the impact of environmental influences on the damage evaluation by its identification or compensation. This might be realized, e.g., by additional sensors for temperature or moisture, by back-calculation of temperature from strain data at an unloaded condition, or by ensuring the unloaded condition by strain data for dynamic evaluations. Besides damage monitoring, self diagnosis should also be addressed in a multi-sensor SHM approach and can be realized by the joint use of different data features. For example, intermediate EMI measurements allow the diagnosis of the PWAS used for the UGW method.

For example, to detect the initiation of a delamination in a CFRP or GFRP component, the combination of the UGW and the EMI method might be promising (cf. Section 3):

- UGW method for far field sensing and SHM Level 4 assessment by linear and nonlinear scattering effects; and
- EMI method for self diagnosis and local SHM Level 3 assessment by linear and nonlinear effects.

In particular, the dynamic EMI and UGW methods are very advantageous to combine as they use the same sensor network. To monitor the further progression of the delamination under operational loads, one can additionally apply methods that have the potential to detect large damage:

- EIT for spatial SHM Level 3 damage assessment if the structure is conductive (CFRP); and
- strain measurements by FOS to reliably assess large blisters

However, additionally sensors for monitoring of environmental influences should be included, as the dynamic methods are strongly influenced by, e.g., moisture and temperature.

### 4.2. Definition of Data Evaluation Procedure

The data evaluation procedure of a multi-sensor SHM approach needs to consider the numerous advantages and disadvantages to most beneficially combine the available measurement data and their method-dependent damage assessment features. Therefore, statistical data on the reliability of the single SHM methods and their damage assessment information are needed to yield a robust final result. To improve the reliability of the combined damage evaluation, a component knowledge database (containing evaluation parameters such as different threshold values or UGW scattering patterns) should be constantly updated during operation and aligned with a centralized knowledge database (a database that includes data over all comparable components under operation).

**Figure 12:**
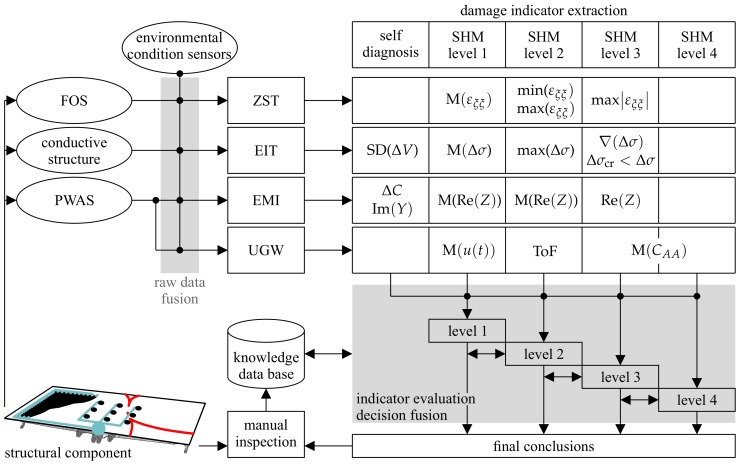
Data evaluation scheme of multi-sensor SHM approach. The illustration shows an aircraft spoiler as the monitored structural component.

The data evaluation process presented in Figure 12 contains four main parts: the sensor network, the raw data fusion, the feature extraction, and the decision making and fusion. The final part yields the conclusions on a potential structural damage. As a consequence of damage detection, manual inspection might be triggered and a knowledge database that governs the decision making process is updated by the findings.

The sensor network depends mainly on the required data for the selected SHM methods. The detailed selection and design of the network is adapted to the structure of interest and its geometrical and environmental constraints. Furthermore, possibly required sensors for the monitoring of uncertain environmental conditions, e.g., temperature, need to be defined. A multi-sensor approach with PWAS transducers, FOS, conductivity sensors, and temperature sensors with corresponding SHM methods, e.g., ZST approach, direct or thin film-based EIT, EMI, and UGW, is expected to allow the assessment of the most relevant damages for both metal and composite structures.

However, the raw measurement data from the sensors need to be associated and fused (model- or data-based) to allow, e.g., temperature or moisture compensation in the damage feature extraction process of the single SHM methods. An example is the model-based temperature and strain raw data fusion for temperature compensation in the monitoring of the neutral axis damage assessment feature of a composite beam [10]. Numerous features and damage indicators for both sensor self diagnosis and the four SHM levels of damage assessment can be extracted (cf. Section 3). The resulting damage indicators are evaluated based on a knowledge database (containing, e.g., threshold values or UGW scattering patterns), which can be model- and/or data-based. Subsequently, the various evaluation results are combined within the different SHM levels by, e.g, a weighted decision method. However, the weighting of the evaluation has to be done carefully and requires information on the sensor network’s condition (self diagnosis) and statistical data on the reliability of the given SHM results. Although various studies addressed the statistical reliability of different SHM methods, the generalization of results remains challenging [24,78,79]. However, appropriate statistical data should contain the reliability of a conclusion (e.g., the probability that true damage location is close to the predicted damage location, as shown in Figure 6b) [24]. The conclusions are drawn subsequently, starting at the detection of a potential damage. If a damage detected (concluded there is one), conclusions on damage localization, size, and type follow in this sequence. Conclusions drawn from prior levels (e.g, damage location) are used for conclusions in higher levels of damage assessment. An example for this stepwise process is the identification of size and type of a damage by UGW scattering patterns, which requires readily knowing the damage location, i.e., from which direction the scattered wave is approaching (cf. Figure 8b) [74]. However, this is not a straightforward process. Conclusions of lower levels can be questioned and corrected if higher assessment levels do not fit. Final conclusions on the health state of a structure (SHM Level 5) trigger manual inspection and repair.

In today’s aircraft design, the sole existence of a damage might require immediate repair according to regulations of the authorities. Future strategies could use a knowledge database or a digital twin (representing structural analysis data) to evaluate the current or predict the further integrity of a given structure to optimally initiate repair according to regulation and safety standards (condition- and prediction-based monitoring) [3,4].

## 5. Conclusions

Single state-of-the-art SHM methods might not be sufficient or adequate for holistic damage assessment of full scale structural components.

The combined application of dynamic SHM methods using PWAS (e.g., EMI and UGW) shows high potential for damage identification due to their sensitivity to many structural properties, but they suffer from structural and environmental uncertainties. Static SHM methods using FOS have a comparatively high technological readiness level and are comparatively simple to back-calculate the actual damage from a damage indicator. Thus, uncertainties can be more easily identified or compensated by additional sensor data. It is furthermore shown that the physical effects used for damage assessment strongly deviate between static and dynamic methods, allowing a more reliable observation due to redundant assessment. Consequently, a reliable multi-sensor SHM approach should combine static and dynamic damage assessment methods. The data fusion for damage evaluation by a multi-sensor approach needs to be adapted to the specific needs of the single methods. Besides SHM methods that readily use data fusion for feature extraction and damage evaluation, the optimal joining of the available data to a reliable damage assessment result is still an open field of research. Therefore, a theoretical, gradual, and clearly arranged data evaluation procedure is proposed that realizes a combined multi-sensor damage assessment based on single state-of-the-art SHM methods.

However, a reliable decision level data fusion requires generalized and verified statistical data, whose development shall be addressed in future research. Furthermore, future multi-sensor approaches should also include structure analysis data to extend today’s damage assessment to a full structure integrity assessment (SHM Level 5).

## Figures and Tables

**Table 1:** Influence of damages on local properties of mechanical structures [14,25,26,27,28]: (+) strong, (∘) average and (−) weak influence.

Material	Damage Type	Material Stiffness *E*	Mass *m*	Damping *c*	Material Conductivity σ	Boundary Formation *a*
metal	notch	∘	−	−	+	+
	crack	+	−	−	+	+
	corrosion	∘	∘	∘	∘	∘
composite	notch	∘	−	−	+	+
	matrix crack	∘	−	∘	∘	−
	fiber crack	+	−	∘	+	−
	delamination	∘	−	∘	∘	+

**Table 2:** Influence of environmental conditions on local properties of mechanical structures [29,30,31,32]: (+) strong, (∘) average, and (−) weak influence.

Material	Influence Type	Material Stiffness *E*	Mass *m*	Damping *c*	Material Conductivity σ	Boundary Formation *a*
metal	temperature	∘	−	−	∘	−
	dirt	−	+	+	−	∘
	electromagnetic radiation	−	−	−	∘	−
	mechanical loads	−	−	−	∘	∘
composite	temperature	+	−	∘	∘	−
	dirt	−	+	+	−	∘
	moisture	+	+	∘	∘	−
	electromagnetic radiation	−	−	−	∘	−
	mechanical loads	−	−	−	∘	−

**Table 3:** Influence of structural properties in SHM methods.

Approach	SHM Method	Sensor	Measurement Entity	Influencing Properties	SHM Level
static	strain sensing	strain gauge	local strain	E,a	2
		FOS	strain along line	E,a	3
	EIT	conductive coating	thin film conductivity	$E,\sigma_{\mathrm{skin}},a$	3
		conductive structure	volume conductivity	$E,\sigma_{\mathrm{vol}},a$	3
dynamic	EMI	piezoelectric element	global EMI	E,m,c,a	3
	guided waves		wave propagation	E,m,c,a	4
	acoustic emission			E,m,c,a	2
